# From Olive Tree to Treatment: Nano-Delivery Systems for Enhancing Oleuropein’s Health Benefits

**DOI:** 10.3390/ph18040573

**Published:** 2025-04-15

**Authors:** Maha Nasr, Salma H. Katary

**Affiliations:** 1 Department of Pharmaceutics and Industrial Pharmacy, Faculty of Pharmacy, Ain Shams University, Cairo 11566, Egypt; 2 Faculty of Pharmacy, Ain Shams University, Cairo 11566, Egypt; salma.hatem19@pharma.asu.edu.eg

**Keywords:** oleuropein, olive leaf, nanocarriers, nutraceuticals, bioavailability

## Abstract

Oleuropein is a natural polyphenolic compound isolated from olive trees (*Olea europaea*). Besides the strong antioxidant effect of oleuropein, it has many pharmacological activities such as anticancer, antidiabetic, anti-inflammatory, antihypertensive, and many other activities. Thus, oleuropein could be used alone or with other drugs to prevent and treat many diseases. Despite its promising health benefits, oleuropein is highly prone to hydrolysis inside and outside the human body, in addition to a poorly identified pharmacokinetic profile and poor bioavailability. Many nanocarrier delivery systems have overcome the delivery limitations of oleuropein in order to maximize its therapeutic benefits. Therefore, this review article sheds light on nano-delivery systems explored until the current date, aiming to enhance oleuropein’s bioavailability and therapeutic impact by improving its pharmacokinetic properties and addressing its stability challenges. Continued research into innovative nanotechnology solutions will be crucial in unlocking the full potential of oleuropein as a powerful nutraceutical and pharmaceutical agent.

## 1. Introduction

**Figure 1:**
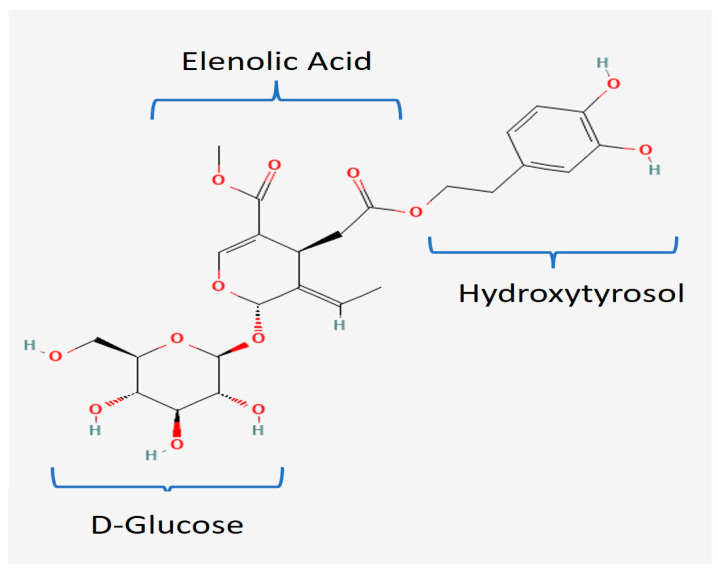
Chemical structure of oleuropein.

The leaves of olive trees have long been known for their valuable properties, since they are a rich source of polyphenolic bioactive compounds [1,2,3]. Oleuropein is the most abundant polyphenolic compound in olive trees (*Olea europaea*); however, it is not exclusively present in olive trees, but also in other plants belonging to the *Oleaceae* family, such as *Syringa*, *Jasminum*, *Phillyrea*, and *Fraxinus* [4,5]. Oleuropein is distributed in many parts of olive trees—leaves, fruit, pulp, and seed—but it concentrates mainly in the unripe fruits and leaves, amounting to approximately 60–90 mg/g leaves dry weight [6,7]. Its yield is variable and is dependent on the climate, genetic makeup, plant cultivar, agriculture practices, harvesting time, collected organs, and extraction technique, among other factors [7,8,9,10]. As shown in Figure 1, oleuropein is an ester between hydroxytyrosol (another olive tree polyphenol) and oleoside (a secoiridoid glucoside of elenolic acid). Its biosynthetic pathway, as a secoiridoid derivative, is the mevalonic acid pathway, and its degradation occurs through enzymatic or chemical hydrolysis into aglycone, hydroxytyrosol, elenolic acid, and glucose, depending on the type of degradation [1,4,10,11]. Because of its proven health benefits, it is available on the market as a whole olive leaf extract [12,13]. Oleuropein is the most abundant compound in olive leaf extract, followed by hydroxytyrosol, the flavone-7-glucosides of luteolin, apigenin, and verbascoside, yielding synergistic therapeutic efficacy. Its therapeutic and preventive activities are mainly attributed to its excellent antioxidant functions [1,5,7,8,14]. Its most highlighted physiological effects are anti-inflammatory, anticancer, antibacterial, antiviral, anti-obesity, antidiabetic, antihypertensive, anti-atherosclerosis, cardioprotective, hepatoprotective, and neuroprotective [5,7,8,10,14], as elaborated in Table 1.

**Figure 2:**
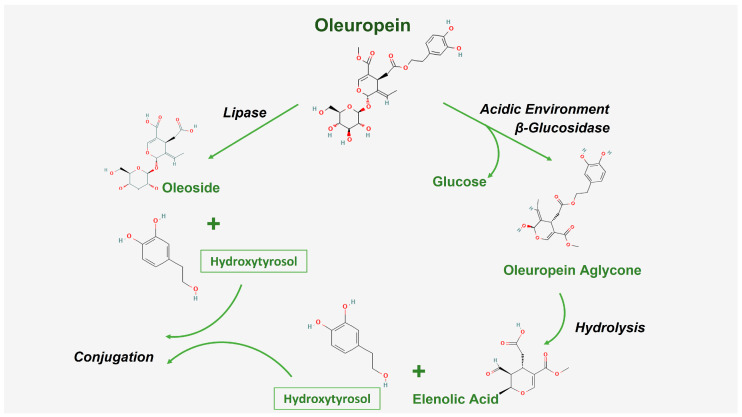
Metabolic pathway of oleuropein.

Despite its aforementioned therapeutic benefits, oleuropein is a challenging molecule for pharmaceutical formulation [20]. It is a large hydrophilic polyphenolic molecule with a molecular weight of 540.5 g/mol and log *p* value −0.4 [5,10]. Oleuropein, like other antioxidants, suffers from many stability challenges, and was reported to be labile to heat, light, and oxygen; thus, it requires mild extraction and formulation steps [10,20]. It also suffers from poor bioavailability, with complex metabolism and a short half-life [5,13,14,19,21,22,23], and is affected by intestinal pH, enzymes, and microbiota [5,14,19,24]. Regarding oleuropein’s absorption, it was reported to undergo acid hydrolysis, forming different metabolites that vary in concentration and distribution depending on the pH and the residence time in the stomach. Being a hydrophilic compound, oleuropein does not readily diffuse through lipid bilayers, but its transport might involve glucose transporters, as hypothesized in the literature. As a consequence, only a small amount of oleuropein reaches the systemic circulation in an intact form [5,25,26]. As shown in Figure 2, oleuropein undergoes extensive metabolism mediated by liver enzymes and gut microbiota, primarily yielding hydroxytyrosol and elenolic acid as major metabolites. The metabolism in the liver occurs through hydrolysis in phase I, producing hydroxytyrosol, which is further conjugated into sulfated or conjugated derivatives in phase II, and unabsorbed oleuropein is subjected to hydrolysis by microbiota in the large intestine, producing more readily absorbed hydroxytyrosol [5,23]. Both oleuropein and its metabolites are reported to be distributed in several tissues in the body, followed by excretion mainly through the renal route [27]. It is worth noting that oleuropein’s bioavailability is also affected by the dose, formulation type, route of administration, age, gender, and food [13,14,19].

Nanotechnology has recently emerged as a promising solution for solving the pharmaceutical challenges of nutraceuticals, among which are carriers such as nanoemulsions, liposomes, nanocapsules, and lipidic and polymeric nanoparticles [28,29,30,31,32]. These nano-delivery systems improve nutraceutical solubility and physical and chemical stability, in addition to attributing controlled and targeted release. They can enhance oral bioavailability by modifying nutraceuticals’ pharmacokinetic profiles, thus directly benefiting their physiological benefits [33,34]. Referring to the aforementioned pharmacokinetic challenges of oleuropein, its formulation in nanoparticles is anticipated to improve its absorption, enhance its stability, and allow for its sustained and targeted release, which would directly reflect on its therapeutic activity.

**Figure 3:**
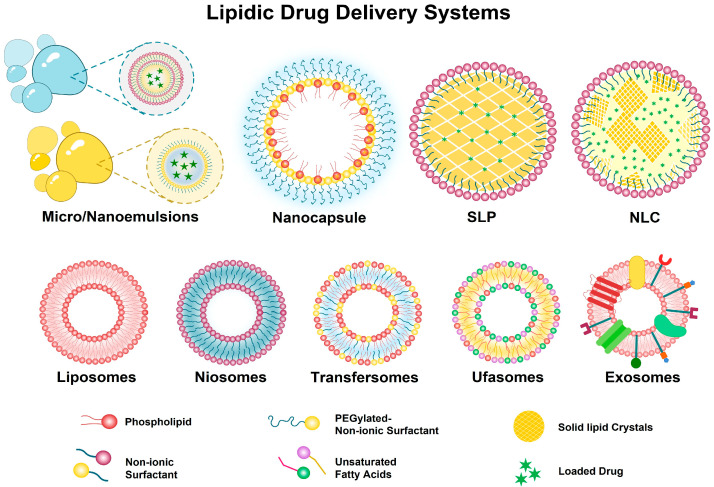
Structural differences between most commonly used nanocarriers.

Therefore, the aim of this review article was to explore the role of nanocarriers in expanding the health benefits of oleuropein by overcoming its pharmaceutical delivery challenges. The structural differences between some of the most commonly used nanocarriers are shown in Figure 3.

## 2. Nanocarriers for Delivery of Oleuropein

### 2.1. Lipid-Based Delivery Systems

Lipidic nano-delivery systems (LNDs) are known for their ability to increase the bioavailability and stability of drugs and control their release, thus leading to greater therapeutic benefits and fewer side effects, in addition to tissue-targeting techniques. LNDs are excellent carriers for hydrophobic drugs, and can also carry hydrophilic drugs with some optimization, with a carrier capacity that can exceed 90%. For oleuropein, emulsions, nanostructured lipidic carriers (NLCs), and lipid nanocapsules have been utilized as potential oleuropein biocompatible nanocarriers, with different aims and routes of administration [35,36].

#### 2.1.1. Nano/Microemulsions

**Figure 4:**
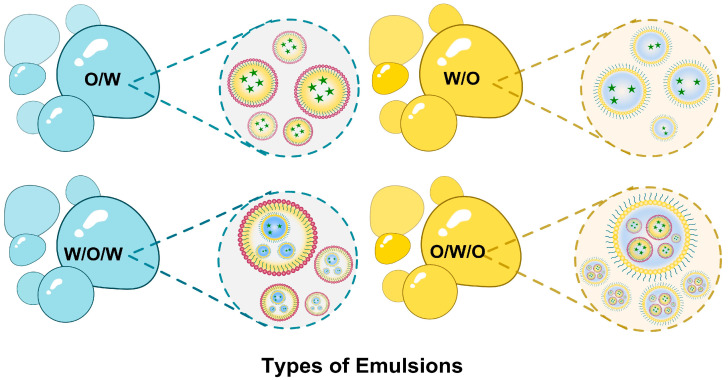
Schematic illustration of the types of emulsions.

Emulsions are lipidic dosage forms composed of a minimum of two immiscible liquid dispersions stabilized by surfactants with or without cosurfactants [37,38]. They are classified according to their physical characteristics into coarse emulsions, nanoemulsions, and microemulsions, with further subclassifications according to their composition into the single emulsion types O/W or W/O and the double or multiple emulsion types W/O/W or O/W/O [39], as demonstrated in Figure 4. Nanoemulsions and microemulsions increase the bioavailability and pharmacological activity of the encapsulated drugs by improving their stability and permeability owing to their nano-size range, as well as their lipidic composition [37,40]. In addition, they exhibit controlled and sustained release for the encapsulated drug and are applicable in various routes of administration [40,41].

A W/O/W nanoemulsion containing oleuropein in the internal aqueous phase to improve its bioavailability and stability and control its release was reported in [42]. This nanoemulsion was made by high-energy methods (homogenization and sonication) using span 80 as the internal W/O interface stabilizer, and pectin with whey protein concentrate (WPC) as the external O/W interface stabilizer. The method and composition were optimized, by response surface methodology, to achieve a double nanoemulsion with a droplet size range of 100–200 nm, zeta potential of −26.8 mV, encapsulation efficacy of 91%, and 1040 µg/mL oleuropein concentration. Oleuropein’s 28-day release behavior was independent of the amount of oleuropein loaded and was responsive to the pH of the emulsion’s external aqueous phase, in which at pH = 6 the WPC–pectin complex formed a double layer on the external O/W interface, resulting in a controlled release of oleuropein. Owing to its promising properties, the same group further optimized the properties of the nanoemulsion using modeling [43].

Oleuropein was also loaded into a W/O microemulsion for topical application at a concentration of 0.2% *w*/*w* to treat psoriasis [44]. The microemulsion consisted of ethyl oleate, transcutol and capryol 90 (surfactant and cosurfactant, respectively), and water, and was prepared using a low-energy emulsification method. A microemulsion of 30.25 nm particle size, neutral surface, and good physical stability for one year at room temperature was prepared. The oleuropein microemulsion exhibited significant penetration and deposition in different skin layers according to an ex vivo study on albino rats’ dorsal skin. In addition, the clinical experiment performed on twenty plaque psoriasis patients delineated the superiority of oleuropein microemulsion over clobetasol propionate, a corticosteroid, through the greater reduction in PASI score (Psoriasis Area and Severity Index) by 70.04% for oleuropein compared with 45.6% for clobetasol, better quality of life, and better dermoscopic alleviation of psoriatic manifestations.

Another oleuropein O/W microemulsion was formulated at a concentration of 0.2% *w*/*w* oleuropein using the water dilution method to potentiate the anticancer effect of oleuropein by enhancing cellular uptake through the small particle size of microemulsions. The microemulsion was composed of oleic acid, tween 20, and ethanol. The results delineated that oleuropein became 160 times more potent as an anti-proliferative drug against HCT-116 colon cancer cells, with IC_50_ of 1.75 µg/mL compared with 185 µg/mL for oleuropein alone. The microemulsion exhibited a droplet size of 10 nm, homogenous dispersion, neutral charge, and physical stability in 3-month ambient conditions. The in vitro release study revealed sustained and complete release of the total oleuropein amount over 24 h [45].

#### 2.1.2. Nanostructured Lipid Carriers (NLCs) and Solid Lipid Nanoparticles (SLPs)

SLPs are composed of solid lipids incorporating the drug, while NLCs are a mixture of solid and liquid lipids, and both are dispersed by a suitable surfactant in an aqueous phase. The incorporation of liquid lipids in NLCs increases the drug-loading capacity of the nanoparticles and enhances their storage stability [46,47,48]. SLPs and NLCs also control drug release profiles, in addition to being biocompatible and lipophilic, which enhance drug bioavailability. They can encapsulate both hydrophilic and lipophilic drugs through optimization of the preparation method and composition [47,49]. Moreover, they are produced on a large scale for cosmetics and food applications and are promising for targeting delivery systems for ocular, dermal, neural, gastric, and pulmonary diseases [48,50].

To the authors’ knowledge, oleuropein has not been encapsulated in SLPs to the present day, but several attempts of its encapsulation within NLCs have been reported (Table 2). Palagati et al. succeeded in entrapping oleuropein in an NLC system for intra-nasal brain targeting through the olfactory pathway to treat bacterial meningitis, thus decreasing the incidence of drug resistance. Through melt-emulsification and then ultrasonication methods and with the help of quality by design (QbD), the oleuropein-loaded NLCs exhibited a small particle size with longer half-life and brain bioavailability. In addition, the oleuropein-loaded NLCs exhibited sustained release over 24 h. The targeting efficacy of oleuropein-loaded NLCs was potentiated as the direct transport percentage was 83.07% when the brain-to-blood ratio was calculated [51]. Another study testing the efficacy of oleuropein-loaded NLCs for meningitis with an in vivo study on Streptococcus pneumoniae-induced meningitis animal model revealed better anti-meningitis activity demonstrated by the reduced neuronal loss and narcotic lesions in the treatment group compared with the untreated group [52]. Huguet-Casquero et al. developed an oleuropein-loaded NLC that could be further optimized as a dry powder for inhalation for the treatment of lung diseases [53] using olive oil as a liquid lipid. The NLC potentiated oleuropein’s antioxidant effect compared with free oleuropein when tested on human lung adenocarcinoma epithelial cells (A549), cystic fibrosis (CuFi-1) cell line, and human normal bronchial epithelial cells (NuLi-1), while exhibiting prolonged release. The same group reported the use of this formulation to treat irritable bowel diseases like acute and ulcerative colitis through oral administration, benefiting from the ability of NLCs to increase the retention time of their payload in inflamed tissues, in addition to their negative charge which could be attracted to the positively charged ulceration proteins, with synergistic anti-inflammatory activity owing to the combination of olive oil (the NLC liquid lipid) and oleuropein (the payload). All in vitro and in vivo results were consistent and confirmed the significantly enhanced ability of oleuropein-loaded NLCs, compared with oleuropein suspension, to reduce TNF-α and IL-6 (pro-inflammatory cytokines), scavenge reactive oxygen species, reduce MPO activity (myeloperoxidase: neutrophils’ lysosomal protein), and keep adequate colon tissue structures [54].

#### 2.1.3. Lipidic Nanocapsules

Lipidic nanocapsules are composed mainly of an oil core surrounded by a pegylated surfactant shell in an aqueous phase [55]. The single-layer surfactant shell has an amount of lecithin phospholipid to increase the stability of the surfactant shell. These nanocapsules are a suitable alternative for liposomes since they are prepared by a solvent-free method and have a longer shelf-life. They have a nano-size range that is highly dependent on their composition and have wide applications in the pharmaceutical field such as passive and active targeting, P-glycoprotein efflux inhibition, gene therapy, and cancer treatment [56].

Regarding oleuropein delivery, Al-Karaki et al. prepared physically stable surfactant-based nanocapsules loaded with oleuropein in their oily cores. They were prepared by the phase inversion method, utilizing Solutol HS15, a surfactant, for the nanocapsule shell. The crafted oleuropein-loaded nanocapsules showed a neutral spherical surface of particle size 151.45 nm. Oleuropein release was complete and sustained over 24 h from the nanocapsules. For the anti-colon cancer activity on the HCT-116 colon cancer cell line, displaying 28 times more anti-colon cancer activity for oleuropein nanocapsules compared with free oleuropein (IC_50_ = 6.52 µg/mL and IC_50_ = 185 µg/mL, respectively) attributed to the enhanced cellular uptake, owing to the small particle size and the surfactant-based shell [57].

### 2.2. Vesicular Systems

Vesicular delivery systems consist of amphiphilic self-assembled molecules which are suitable for both hydrophilic and hydrophobic drugs [58]. They are widely used owing to their advantages such as nano-size, biocompatibility, targeted drug delivery, controlled release, and increased drug stability. Vesicular nanoparticles reported for oleuropein encapsulation include liposomes, niosomes, transfersomes, ufasomes, and hybrid exosomes. They all have the same general structure but different building units, and thus have variable properties and uses [59]. Liposomes, the first developed nanovesicular system, comprise mono- or multi-phospholipid bilayers with an aqueous core. Niosomes have the main structure of liposomes but are made up of non-ionic surfactants, resulting in better stability and entrapment efficacy and lower cost than liposomes. Transfersomes consist of both phospholipids and surfactants, making them more deformable and more suitable for topical and transdermal delivery than liposomes and niosomes. Ufasomes are unsaturated fatty acid liposomes, in which the combination of unsaturated fatty acids and phospholipids increase their stability and drug entrapment efficacy. Lastly, exosomes are a type of extracellular vesicle that have recently been a focus for many researchers due to their excellent targeting abilities and safety [60,61,62]. A summary of oleuropein vesicular systems is shown in Table 3.

#### 2.2.1. Liposomes

Oleuropein-loaded liposomes composed of DOPE (1,2-dioleoyl-sn-glycero-3-phosphoethanolamine) and DOPC (1,2-dioleoyl-sn-glycero-3-phosphocholine) were prepared by a thin-film hydration method, displaying potentiated anti-inflammatory and antioxidant properties of oleuropein when tested on human chondrocytes, suggesting their promising use in the treatment of osteoarthritis [63]. Nassir et al. prepared surface-functionalized folate-pegylated (PEG) liposomes loaded with oleuropein administered by intravenous administration for treatment of prostate cancer [64]. The results showed sustained release and remarkable anti-prostate cancer activity compared with an oleuropein solution, attributed to passive and active targeting, in which the suitable particle size of liposomes allowed for enhanced permeability and retention (EPR), the PEG coating allowed the evasion of the reticuloendothelial system (RES) for passive targeting, and the folate decoration allowed for active targeting. Similarly, PEG-phytosomes were developed for passive targeting of oleuropein in combination with rutin in colon cancer, resulting in the potentiated anticancer effect of oleuropein [70].

#### 2.2.2. Niosomes

Oleuropein-loaded niosomes were explored for their ability to prevent and treat breast cancer brain metastasis cases [66]. Niosomes were prepared from tween 60 and cholesterol to increase oleuropein distribution into the brain parenchyma and to cross the blood–brain barrier (BBB). They exhibited good storage stability at 25 °C and 4 °C for up to 50 days, in addition to increased cellular uptake and pH-sensitive sustained release triggered by the tumor’s acidic microenvironment.

#### 2.2.3. Transfersomes

Oleuropein transfersomes were mainly developed for topical administration, to enhance oleuropein permeation and deposition in skin layers, and thus enhance therapeutic effects for skin-related diseases. Sklenarova et al. co-loaded oleuropein with lentisk oil in tween 80-modified liposomes (transfersomes), hyaluronate-modified liposomes (hyalurosomes), and their combination (hyalutransferosomes) [67]. The three systems exhibited sustained release over 24 h, with enhanced stability of vesicles upon freeze-drying with mannitol as a cryoprotectant, without affecting the stability of oleuropein. In another study, Allaw et al. modified collagen-enriched liposomes with tween 80 (collagen transfersomes), glycerol (collagen glycerosomes), and both tween 80 and glycerol (collagen glytransfersomes) [68]. Physical stability was demonstrated by the systems during 4 months’ storage time, and collagen glytransfersomes displayed promising wound-healing properties.

#### 2.2.4. Ufasomes (Unsaturated Fatty Acid Liposomes)

Cristiano et al. reported oleuropein delivery by ufasomes, made up of unsaturated fatty acids (oleic and linoleic acids) with or without Phospholipon^®^ 90G (a phospholipid) [69]. Ufasomes that included phospholipids in their bilayers were better than ufasomes containing only fatty acids regarding the entrapment of oleuropein, shelf stability, and biocompatibility. The composition of oleic acid, linoleic acid, and Phospholipon^®^ 90G (molar ratio 1:1:0.5) yielded ufasomes with high negative charge and considerable physical stability. The unsaturated fatty acids and lecithin bilayer enhanced the cellular permeation and the pharmacological antioxidant and anti-inflammatory properties of oleuropein.

#### 2.2.5. Exosome–Liposome Hybrids

Aliakbari et al. used the freeze–thawing hybridization method to develop a novel vesicular delivery system for oleuropein against neurodegenerative diseases such as Parkinson’s disease [71]. This novel system is a mesenchymal stem cell exosome hybridized with zwitterionic liposomes. The exosomal hybridization conferred more advantages to this system over conventional liposome-loaded oleuropein, such as enhanced cell internalization, better BBB permeability, and better interaction with α-synuclein protein fibrillation, resulting in fibrillation inhibition and depolymerization of mature fibrils.

### 2.3. Polymeric Delivery Systems

Polymeric delivery systems are known for their increased stability, and their ability to enhance the bioavailability of nutraceuticals. The polymers used can either be from a synthetic or natural source, such as carbohydrates and proteins [72]. Several polymers such as cyclodextrin, chitosan, and polylactic acid have been reported to encapsulate oleuropein.

#### 2.3.1. Cyclodextrin-Based Nanoparticles

Cyclodextrin is a biocompatible and biodegradable glucose oligomer, and is classified into three types: α, β, and γ, made up of 6, 7, and 8 α-glucopyranose units, respectively. The glucose units are linked together through α-1,4 glycosidic linkage to form a truncated circular cone 3D shape with a hollow hydrophobic core and external hydrophilic surface. The narrow rim of the cone 3D shape is called the primary side, while the wide rim is the secondary side of cyclodextrin. The core is suitable for inclusion, by complexation, of hydrophobic organic payloads with suitable size resulting in a stable complex with a high equilibrium constant, thus increasing the payload’s aqueous solubility and stability. The relative hydrophilicity and aqueous solubility of cyclodextrin were attributed to the sugars’ hydroxyl groups on the outer surface which could be further improved through the hydroxyl groups’ chemical modification [73,74].

Burgalassi et al. reported ophthalmic drops composed of oleuropein complexed in hydroxypropyl-β-cyclodextrin and encapsulated inside a liposomal vesicle to treat the dry eye syndrome, by virtue of the antioxidant and proliferative activities of oleuropein [75]. The solubility of the oleuropein–cyclodextrin complex allowed its positioning in the hydrophilic liposomal core instead of the liposomal bilayer as in [63]. The oleuropein–cyclodextrin complex was prepared using the precipitation method to be encapsulated inside conventional-type liposomes using the thin-film hydration method. The selected formulation exhibited a particle size of 235.5 nm, entrapment efficacy of 80.77%, and high physical stability. However, in in vitro testing on rabbit corneal epithelial cell lines, the ability of oleuropein-in-cyclodextrin-in-liposome (prepared in the physiologic pH) to increase cell proliferation and free radical scavenging was less than the free oleuropein, which the authors attributed to the sustainment of oleuropein release, being encapsulated in a dual system: the β-cyclodextrin and the liposomal vesicle.

#### 2.3.2. Chitosan Nanoparticles

Chitosan is a natural linear polycationic polysaccharide consisting of two types of randomly ordered monomers, D-glucosamine and N-acetyl-D-glucosamine. The degree of deacetylation and molecular weight determine many of chitosan’s properties like degradability, mechanical strength, and drug encapsulation efficacy. Colloidal chitosan nanoparticles enhance drug solubility and control its release. Because of the cationic nature of chitosan, it has mucoadhesive properties leading to enhanced loaded drug bioavailability, delineating chitosan nanoparticles as a promising delivery system for several diseases as a drug carrier for many diseases [76].

Wang et al. and Zhao et al. prepared oleuropein-loaded chitosan nanoparticles, using the ionotropic gelation method, to be used as a dentin primer/adhesive [77,78]. In the field of adhesive dentistry, resin composite is needed to repair tooth defects, but the bonding strength between the resin and teeth matrix is weak and unpredictable, especially for dentin–resin bonding, and dentin primers are needed to enhance the dentin–resin bonding. The oleuropein–chitosan nanoparticles were able to crosslink with dentin’s collagen through hydrogen bonds in a dose-dependent manner to protect the exposed collagen from matrix metalloproteinase and other environmental proteases (e.g., in saliva). Crosslinking improved dentin mechanical properties and resulted in an aging- and biodegradation-resistant dentin–resin bonds. Oleuropein–chitosan nanoparticles were delineated as a potential dentin primer with anti-microbial and prolonged restoration, displaying a particle size of 161.29 nm, zeta potential of 19.53 mV, and biphasic release pattern (burst then sustained release), in addition to enhanced oleuropein stability.

In another study, the encapsulation of oleuropein inside chitosan was reported to potentiate oleuropein’s protective anti-inflammatory and antioxidant activities against ethanol-induced gastric ulcers [79]. The ionotropic gelation method was used to prepare oleuropein-loaded chitosan nanoparticles, displaying values of 174.3 nm, +11.2 mV, and 92.81% in particle size, surface charge, and encapsulation efficacy, respectively. The release pattern was biphasic in simulated gastric conditions; immediate release (20% of oleuropein in 1 h) then sustained for 8 h. The enhanced in vivo behavior of oleuropein–chitosan nanoparticles was ascribed to the mucoadhesive nature of the nanoparticles caused by electrostatic binding of the negatively charged gastric mucin and the positive charges of chitosan amino groups that were further protonated in the acidic gastric pH.

#### 2.3.3. Nanofibers

Nanofibers are a polymeric system with a fiber diameter in the nanometer range, and calculated high surface area and porosity. Nanofibers were developed mainly for site-controlled drug release. The most used preparation method for nanofibers is electrospinning, used for wound dressings and implants, in addition to their applications in oral and transdermal delivery [80,81].

An application for oleuropein as a potential local therapy for recurrent glioblastomas (GBs) after surgical excision was investigated [82]. Oleuropein-loaded polylactic acid nanofibers were prepared using coaxial (core–shell) electrospinning to control oleuropein release over time through encapsulation inside the polymer nanofiber shell. The nanofibers exhibited an average fiber diameter between 133 and 139 nm and beaded morphology directly related to the oleuropein solvent viscosity. Oleuropein release was time-dependent with maximum release at 72 h. In addition, the controlled oleuropein release was adversely affected by the thickness of the web, i.e., more ingredients in the higher-thickness areas. The results showed a significant reduction in cell viability on a fibroblast-like GB cell line (T98G) from 52.9% in the first 24 h to 13.6% after 120 h. This linear decrease in cell viability is related to the increasing oleuropein release from the nanofibers with time compared with only initially (first 24 h) reduced cell viability in cells treated with oleuropein only. Co-loading of oleuropein with temozolomide (post-surgical standard therapy for glioblastoma) in polylactic acid nanofibers induced additive effects through prolonged exposure to oleuropein and temozolomide together. This combination led to more reduction in growth rates and tumor cell invasion, more apoptosis induction, and more sphere-size reduction in the 3D glioblastoma spheroid model.

In another study, a pentadecalactone–valerolactone enzymatically synthesized copolymer (PDL-VL) was blended with gelatin, a natural polymer, to produce a nanofibrous wound dressing loaded with oleuropein [83]. Nanofibers were prepared using electrospinning followed by crosslinking with glutaraldehyde, displaying defect-free surface and a fiber diameter ranging from 560 to 806 nm that was directly proportional to oleuropein content. Crosslinking increased the water and thermal resistance of the oleuropein-loaded nanofiber, resulting in a more durable wound dressing that resists degradation. Interestingly, oleuropein was also reported to act as a crosslinking agent, so it is anticipated that it would have enhanced crosslinking together with glutaraldehyde [84]. The antimicrobial and cytotoxicity tests proved the potential benefits of 75% *w*/*v* oleuropein-loaded PDL-VL/gelatin nanofiber as a biocompatible wound dressing with protective antimicrobial activity against both Gram-positive and Gram-negative skin pathogens.

Furthermore, another nanofiber made of silk fibroin by electrospinning was prepared, and further loaded with oleuropein, to be used as an antimicrobial wound dressing [85]. The oleuropein-loaded nanofiber had an average diameter of 92 nm, with a smooth and high external surface area, leading to an adsorption capacity of 228.34 mg oleuropein per gram nanofiber, compared with the prepared silk fibroin microfibers. Oleuropein conferred antibacterial properties on the nanofibers, delineating them as a promising wound-healing system, with proven enhancement of cell migration.

#### 2.3.4. Inorganic Nanoparticles

Metallic nanoparticles (MNPs) are well reported in the diagnosis and treatment of many diseases, owing to their antioxidant, anticancer, antiviral, and antimicrobial properties. MNPs have many advantages, such as nano-size, increased drug bioavailability, variable possible coatings, tissue targeting, reduced drug side effects, controlled drug release, and increased aqueous solubility of hydrophobic drugs. The biosynthesis of MNPs using plant materials, depending on the plant’s secondary metabolites, has been the interest of many researchers because of its low cost, speed, and green nature [86]. Both oleuropein and the whole leaf extract were reported to aid in the synthesis of colloidal metallic nanoparticles (silver, gold, copper, and iron) by acting as reducing agents to convert the metal from the ion form (X^+^) to the neutral form (X^0^). This green approach for synthesizing metallic nanoparticles improves their medical properties [87], safety [88], and stability [89].

Magnetic nanoparticles loaded with oleuropein were prepared to treat gastric adenocarcinoma [90]. Iron nanomagnetite was co-precipitated in a stoichiometric ratio of 1:2 for Fe_2_ and Fe_3_, respectively, then covered with silica and further functionalized with 3-Aminopropyl Trimethoxysilane (3-APTMS). Oleuropein’s carboxylic acid groups were bonded to the free amino groups of APTMS through amide bonding, forming so-called NH_2_-connected bridges. The nanomagnetic oleuropein, with a size range of 39.50 to 73.57 nm, had a significant dose- and exposure-time-dependent proliferation inhibition of the gastric adenocarcinoma cell line, delineating it as a promising physical means of targeting oleuropein by an external magnetic field. Other studies used oleuropein-loaded iron oxide (Fe_3_O_4_) nanoparticles for colorectal cancer therapy [91,92]. Magnetic iron oxide was first prepared, then coated with glucose as a ligand and conjugated with oleuropein. The synergistic effect of iron oxide, glucose, and oleuropein was reflected by the increased colorectal cancer cell (SW480) apoptosis and reduced proliferation, invasion, and metastasis through inhibiting long non-coding RNA associated with the KRAS pathway and inhibiting the KRAS-influenced genes. This oleuropein-loaded iron nanoparticle had an average size of 26–76 nm, and an average zeta potential of −21.6 mV, in addition to having good physical and thermal stability, and could be used through physical targeting with an external magnetic field as a local cancer-targeting moiety.

The well-established antioxidant activity of both oleuropein and silver nanoparticles was combined in a single delivery system to prevent doxorubicin-induced testicular damage [93]. The accumulation of reactive oxygen species mediates this damage through the increased pro-inflammatory cytokines and pro-apoptotic proteins, which leads to reduced sperm count and motility and abnormal sperm morphology. Silver nanoparticles were prepared through a reduction reaction of silver ions with sodium citrate, which acted as a reductant and stabilizer, and provided functional groups for electrostatic interaction with oleuropein. Oleuropein-loaded silver nanoparticles, with an average particle size of 10–60 nm, counteracted doxorubicin-induced testicular toxicity, as observed in in vivo and histopathological tests.

Mesoporous silica nanoparticles are biodegradable, biocompatible, and safe non-metallic nanoparticles with adjustable pore size and distribution, serving as a cavity for loaded drugs leading to their controlled release [94]. Oleuropein-loaded mesoporous silica nanoparticles were developed by Park et al. as a promising treatment for 5-fluorouracil-resistant colorectal cancer [95]. Silica was loaded over a surfactant’s self-assembled hexagonal array that was subsequently dissolved and substituted with Zn and Au ions inside. The oleuropein-loaded ZnO/Au mesoporous silica nanoparticle had a spherical monodispersed size distribution with an average particle size of 240.5 nm and relatively low average zeta potential of −12.0 mV that contributed to the stability of the nanoparticles owing to the incorporated positive metallic ions. This delivery system potentiated oleuropein effects, since comparable results to free oleuropein were obtained although oleuropein content in the mesoporous nanoparticles was 2.5-fold lower. The in vitro investigations on 5-FU-resistant DLD-1 cells showed biocompatibility, three times higher induced late-phase apoptosis, reduced cellular proliferation, reduced invasion and migration, reduced colony-forming ability, augmented reactive oxygen species accumulation, and mitochondrial dysfunction.

## 3. Conclusions

Oleuropein, a major bioactive component of olive leaves, has been widely recognized for its diverse pharmacological properties, including antioxidant, anti-inflammatory, antimicrobial, cardioprotective, and neuroprotective effects. Its potential therapeutic applications in metabolic disorders, neurodegenerative diseases, cardiovascular conditions, and even cancer have drawn significant research interest. However, despite its promising benefits, several challenges remain that limit its full clinical potential. One of the primary challenges associated with oleuropein is its low bioavailability. Rapid metabolism and poor absorption hinder its therapeutic efficacy, necessitating the development of advanced delivery systems such as nanoformulations. Another significant challenge is the need for robust clinical evidence. While numerous in vitro and animal studies have demonstrated oleuropein’s pharmacological effects, human clinical trials remain limited and often suffer from small sample sizes and inconsistent methodologies. More well-designed, large-scale clinical studies are needed to validate its safety, optimal dosing, and long-term effects. Future research should focus on addressing these limitations by enhancing formulation strategies to improve bioavailability, establishing standardized extraction protocols, and conducting rigorous clinical trials to confirm therapeutic efficacy. Additionally, exploring synergistic interactions with other bioactive compounds may enhance oleuropein’s pharmacological potential.

## Figures and Tables

**Table 1:** The crucial health benefits of oleuropein.

Disease	Mechanism of Action	Ref.
Cancer	Inhibition of cancer cell growth and viability Inhibition of cell motility and invasion Induced apoptosis Antiproliferative effect Cell-cycle arrest Oxidative stress suppression Potentiation of concomitant anticancer drugs Anti-angiogenic activity	[5,15,16]
Obesity	Adipogenesis regulation Increased satiety Increased oxygen consumption Increased plasma catecholamines	[5,8,10]
Diabetes	Promotes glucose-stimulated insulin secretion Increases glucose cellular uptake Reduction in amylin–amyloid-induced cytotoxicity Competition with glucose on absorption sites Inhibition of gut α-amylase and α-glucosidase Regulates gut microbiota	[5,17]
Atherosclerosis	Membrane lipid peroxidation inhibition Up-regulation of liver LDL receptor HDL, LDL, cholesterol, and triglyceride regulation Anti-inflammatory and oxidative stress suppression Cell adhesion reduction	[5,15,18]
Alzheimer	Reduction in amyloid β formation and aggregation and plaque deposition Tau protein inhibition Reduction in neuronal inflammation and toxicity	[5,15,18,19]
Parkinson	α-Synuclein amyloid genesis inhibition Lower reactive oxygen species production Increased antioxidant enzymes Decreased neuronal loss	[5,15,18]

**Table 2:** Properties of oleuropein NLC delivery systems.

Composition (Solid and Liquid Lipids)	PS (nm)	ZP (mV)	EE (%)	Status of Investigation	Main Findings	Ref.
Tefose^®^ and Capmul^®^	169.5	−27	98.4	- In vitro hemolytic assay - Histopathology on goat nasal mucosa - In vivo pharmacokinetic study on albino Wistar rats	- Hemocompatibility with blood components - Biocompatibility with nasal mucosa - Prolonged half-life and greater brain bioavailability compared with OL solution, thus reduced dose and frequency	[51]
Precirol^®^ ATO 5 and Campul^®^	357.8	−40.1	53.42–89.4	- In vivo anti-meningitis activity in bacteria-induced meningitis in albino Wistar rats	- Histopathological examination revealed a significantly smaller area in neural loss in addition to the regeneration of brain tissue in the hippocampus region in the OL-loaded NLC-treated group	[52]
Precirol^®^ ATO 5 and olive oil	150	−21	99.12	- Radical scavenging activity by the DPPH assay - In vitro CAA assay on three lung epithelial cell lines	- Enhanced antioxidant power of OL-loaded NLCs over OL alone - Significant dose-dependent alleviation of oxidative stress	[53]
Precirol^®^ ATO 5 and olive oil	141.2 ± 12.3	−25.3 ± 2.7	99.95	- In vitro assays on J774 murine macrophages - In vivo efficacy study on acute DSS colitis male C57BL/6 mice model	- Safe and biocompatible delivery system - Reduced TNF-α levels - Higher ROS scavenging activity - Lower MPO activity - More normal colon cellular architecture	[54]

Cellular antioxidant activity (CAA), radical scavenging activity assessment by 2,2-diphenyl-1-picrylhydrazyl (DPPH), dextran sodium sulfate (DSS), entrapment efficacy (EE), myeloperoxidase (MPO), nanostructured lipid carrier (NLC), oleuropein (OL), particle size (PS), reactive oxygen species (ROS), tumor necrosis factor type alpha (TNF-α), zeta potential (ZP).

**Table 3:** Properties of oleuropein vesicular delivery systems.

Composition	PS (nm)	ZP (mV)	EE (%)	Status of Investigation	Main Findings	Ref.
Zwitterionic liposomes	106.1 ± 7	−19.9 ± 9.7	30.2 ± 1.6	- In vitro cytotoxicity assay on NIH3T3 mouse fibroblast - In vitro cytocompatibility assay on human chondrocytes	- OL-loaded liposomes showed no cytotoxicity against fibroblasts at any concentration - OL-loaded liposomes were delineated as an effective delivery system to human chondrocytes with direct release into the cells	[63]
Surface functionalized folate PEG-liposomes	184.2 ± 9.16	1.41 ± 0.24	63.52 ± 4.15	- In vitro activities on 22Rv1 cells (MTT, PS externalization, TUNEL, mitochondrial membrane potential, and caspase-3 assays) - In vivo assays on male BALB/c nude mice	- Inhibited cell viability with IC_50_ of 132.23 µM - Significantly higher cell apoptosis than OL solution - Six-fold increase in the AUC (bioavailability) compared with OL solution - Significantly higher rates of tumor growth inhibition, more resistance to body weight change, and higher survival probability	[64]
Liposomes	(BFD) 115 ± 3.2 (AFD) 165.4 ± 1.4	(BFD) 23.1 ± 1.2 (AFD) 15.8 ± 0.2	50–60	- In vitro release assay under stimulated intestinal fluid (SIF) - In vitro ABTS·+ and DPPH antioxidant assays	- First-hour release of 18% of oleuropein content, with non-Fickian diffusion - Enhanced antioxidant activity compared with oleuropein alone	[65]
pH-sensitive niosomes	85 ± 7.381	1.38 ± 0.074	96.89 ± 0.241	- In vitro MTT cytotoxicity and cellular uptake assay on 4T1 cancer cells - In vivo assay on female Wistar rats with breast cancer brain metastasis	- Potentiated OL cytotoxicity with 92.67 µg/mL IC_50_ and cytocompatibility on normal cells - Increased cell internalization and confirmed pH-sensitive release based on FITC dye intensity - Two-fold increase in OL accumulation in brain tissue - Prolonged survival rate	[66]
Transfersomes	(BFD) 141 ± 19 (AFD) 149 ± 10	(BFD) −65 ± 5 (AFD) −63	(BFD) 88 ± 1 (AFD) 67 ± 2	- In vitro scratch assay on primary normal human dermal fibroblasts - ELISA for quantification of MMP-1, MMP-2, IL–6 and IL-8	- Higher cell proliferation and migration to the affected area - 74% scratch closure at 24 h with the highest dilution of 0.2 µg/mL oleuropein, compared with 50% closure for the untreated scratched cells - Significant reduction in MMP-1 and IL-6, while lower effects in reducing MMP-2 and IL-8	[67]
Hyalurosomes	(BFD) 146 ± 25 (AFD) 104 ± 6	(BFD) −64 ± 2 (AFD) −59	(BFD) 90 ± 2 (AFD) 67 ± 5			
Hyalutransfersomes	(BFD) 153 ± 22 (AFD) 101 ± 11	(BFD) −63 ± 6 (AFD) −67	(BFD) 89 ± 1 (AFD) 68 ± 2			
Collagen glytransfersomes	113 ± 6	−41 ± 9	92 ± 7	- In vitro MTT cytotoxicity assay - Scratch wound assay	- High biocompatibility and complete restoration of healthy skin fibroblasts after being treated with H_2_O_2_ - Impressive reduction in nitric oxide release - Almost complete scratch closure after 48 h	[68]
Ufasomes	199 ± 1	−42 ± 1	89 ± 2	- In vitro MTT and LDH assays on CaCo-2 cells	- Good biocompatibility and enhanced protection against oxidative stress caused by H_2_O_2_	[69]

After freeze-drying (AFD), 2,2′-azino-bis(3-ethylbenzothiazoline-6-sulfonic acid assay (ABTS·), before freeze-drying (BFD), radical scavenging activity assessment by 2,2-diphenyl-1-picrylhydrazyl (DPPH), entrapment efficacy (EE), enzyme-linked immunosorbent assay (ELISA), fluorescein isothiocyanate (FITC), interleukin (IL-6, -8), lactate dehydrogenase (LDH), 3-[4,5-dimethylthiazol-2-yl]-2,5 diphenyl tetrazolium bromide assay (MTT), matrix metalloproteinases (MMP-1, -2), oleuropein (OL), particle size (PS), zeta potential (ZP).
